# Unleashing the Power of Multiomics: Unraveling the Molecular Landscape of Peripheral Neuropathy

**DOI:** 10.1002/acn3.70019

**Published:** 2025-03-24

**Authors:** Julie Choi, Zitian Tang, Wendy Dong, Jenna Ulibarri, Elvisa Mehinovic, Simone Thomas, Ahmet Höke, Sheng Chih Jin

**Affiliations:** ^1^ Department of Genetics School of Medicine, Washington University St. Louis Missouri USA; ^2^ Department of Neurology, Neuromuscular Division Johns Hopkins University School of Medicine Baltimore Maryland USA; ^3^ Department of Neuroscience Johns Hopkins University School of Medicine Baltimore Maryland USA; ^4^ Department of Pediatrics School of Medicine, Washington University St. Louis Missouri USA

## Abstract

Peripheral neuropathies (PNs) affect over 20 million individuals in the United States, manifesting as a wide range of sensory, motor, and autonomic nerve symptoms. While various conditions such as diabetes, metabolic disorders, trauma, autoimmune disease, and chemotherapy‐induced neurotoxicity have been linked to PN, approximately one‐third of PN cases remain idiopathic, underscoring a critical gap in our understanding of these disorders. Over the years, considerable efforts have focused on unraveling the complex molecular pathways underlying PN to advance diagnosis and treatment. Traditional methods such as linkage analysis, fluorescence in situ hybridization, polymerase chain reaction, and Sanger sequencing identified initial genetic variants associated with PN. However, the establishment and application of next‐generation sequencing (NGS) and, more recently, long‐read/single‐cell sequencing have revolutionized the field, accelerating the discovery of novel disease‐causing variants and challenging previous assumptions about pathogenicity. This review traces the evolution of genomic technologies in PN research, emphasizing the pivotal role of NGS in uncovering genetic complexities. We provide a comprehensive analysis of established genomic approaches such as genome‐wide association studies, targeted gene panel sequencing, and whole‐exome/genome sequencing, alongside emerging multiomic technologies including RNA sequencing and proteomics. Integrating these approaches promises holistic insights into PN pathophysiology, potentially revealing new biomarkers and therapeutic targets. Furthermore, we discuss the clinical implications of genomic and multiomic integration, highlighting their potential to enhance diagnostic accuracy, prognostic assessment, and personalized treatment strategies for PN. Challenges and questions in standardizing these technologies for clinical use are raised, underscoring the need for robust guidelines to maximize their clinical utility.

## 1 Introduction

**FIGURE 1:**
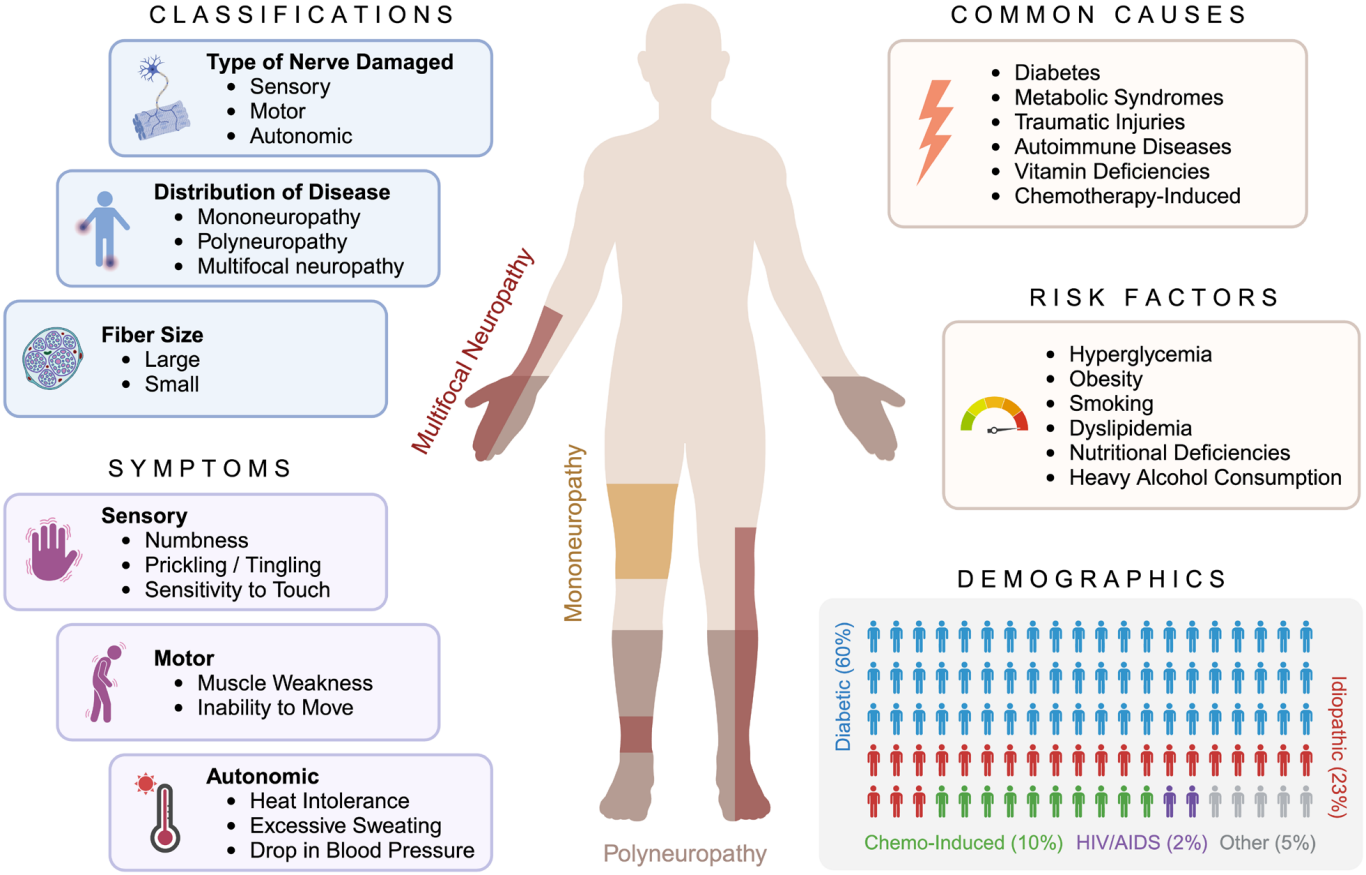
Clinical aspects of PN. The overview of clinical aspects of PN highlights general classification criteria, typical symptoms, common causes, risk factors, and demographic breakdown by type.

Peripheral neuropathies (PN) are debilitating conditions caused by damage to the peripheral nerves, with symptoms varying by nerve type and severity. Patients may experience sensory disturbances like numbness and tingling, motor impairments that cause muscle weakness, and autonomic dysfunctions affecting temperature regulation, sweating, and blood pressure control [1]. The etiology and mechanism of PN are diverse, each associated with different pathological processes (Figure 1) [2]. Most PN cases are secondary to existing or acquired health conditions, such as diabetes or metabolic syndromes. Hereditary PN, though less common, is caused by genetic variants that impair nerve function or structure. Among these, Charcot–Marie–Tooth (CMT) disease is the most prevalent inherited neurological disorder, characterized by clinical and genetic heterogeneity with more than 130 causal genes linked to various subtypes of the disease [3, 4, 5]. Accurate diagnosis of PN is critical due to its diverse etiologies and clinical presentations. Current diagnostic tools for PN include familial medical history, neurological examination, nerve conduction studies, blood testing, clinical imaging, biopsy, and gene panel testing for known hereditary PN genes [6]. In particular, genetic testing has been instrumental in determining common CMT subtypes caused by *PMP22* duplications (CMT1A), *GJB1* variants (CMTX1), *MPZ* variants (CMT1B), and *MFN2* variants (CMT2A) [6, 7]. Despite extensive investigations, approximately 25%–46% of all PN cases have unknown etiology and are termed idiopathic PN [3].

The clinical understanding of hereditary PN may trace its early roots to 1852, when Auguste Nélaton first observed peripheral neuropathy with hereditary perforating foot ulcers [8]. In 1886, Jean‐Martin Charcot, Pierre Marie, and Howard Henry Tooth independently described cases of sporadic and familial hereditary motor and sensory neuropathy, leading to the condition's name: Charcot–Marie–Tooth disease [9]. Early genomic studies utilized conventional techniques, such as pedigree linkage analysis and recombinant DNA methodologies, to investigate and classify PN subtypes. For example, linkage mapping proved crucial in identifying key linkages on Chromosomes 1 and 17, leading to the discovery of the 1.5 Mb tandem duplication of chromosome 17p11.2–p12 encompassing the *PMP22* gene, which occurs in 60%–70% of CMT1 patients [10, 11, 12, 13]. This discovery became one of the first genetic examples of submicroscopic genomic disorder.

The mid to late‐20th century witnessed significant advances in cytogenetic and molecular genetics, including techniques such as fluorescence in situ hybridization (FISH), polymerase chain reaction (PCR), and Sanger sequencing [14, 15]. The combination of PCR and Southern blotting techniques facilitated the discovery of multiple genes associated with PN, including *LIMP‐2* with complex neurological disorders, including peripheral demyelinating neuropathy, and *SLC12A6* with agenesis of the corpus callosum with PN, in both murine and human models [16, 17, 18]. Sanger sequencing, in particular, remains a gold standard for confirmatory clinical applications and is essential for validating advanced genomic technologies, given its precision in identifying nucleotide changes in target genes [19]. Protein‐based methods, such as Western blotting, have also proven instrumental in characterizing proteins and antibodies linked to inflammatory and immunologically associated PN subtypes, revealing key PN pathogenic factors such as mitochondrial dysfunction and anti‐CV2 antibodies [20, 21].

However, the true paradigm shift in PN genomic research emerged with next‐generation sequencing (NGS) during and after the Human Genome Project. NGS has revolutionized clinical genetic diagnostics by dramatically enhancing DNA sequencing performance, uncovering novel disease‐causal variants, variants of unknown significance [22], and providing insights into the heterogeneity of variant distribution in PN [23]. Notably, CMT research was the first in medicine to use whole‐genome sequencing (WGS) for identifying clinically relevant variants and providing precise diagnostics, underscoring the transformative impact of NGS on PN research and treatment [24].

**FIGURE 2:**
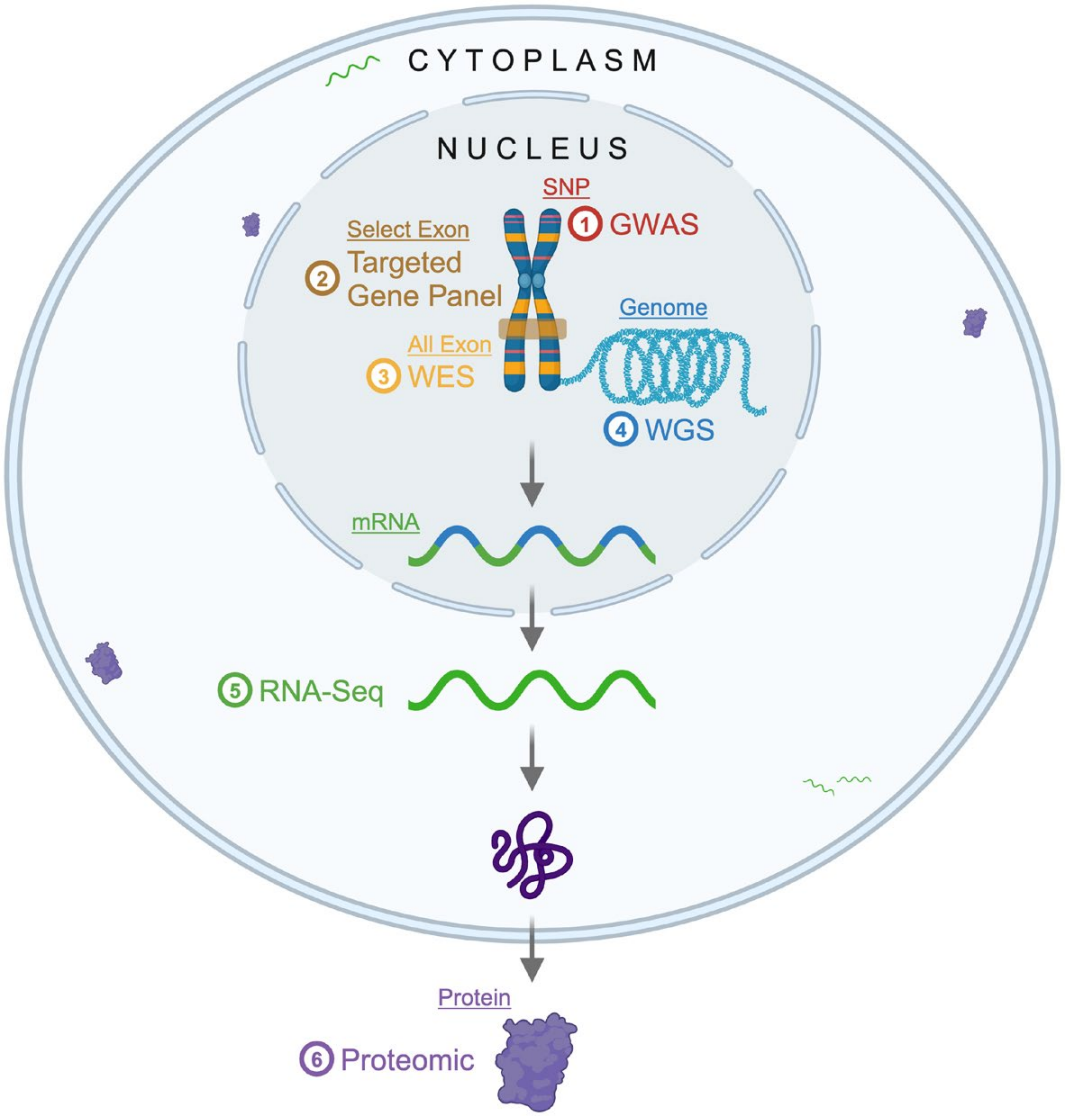
Cellular view of multiomic technologies. The simplified schematic illustrates cellular components related to the technologies described in the review.

This review explores the rapid advancements in genomic technologies in the early 21st century following the Human Genome Project. It examines the advantages and limitations of PN research using NGS, including genome‐wide association studies (GWAS), targeted gene panel sequencing, whole‐exome sequencing (WES), WGS, and long‐read sequencing (LRS). Additionally, it highlights emerging multiomic technologies, such as Proteomics, and their roles in advancing genomic medicine and enhancing PN research (Figure 2).

## 2 Multiomic Technologies

### 2.1 GWAS

Since the early 2000s, GWAS have revolutionized genomic research by enabling exploration into diverse genotype–phenotype associations across a broad spectrum of complex traits and diseases [25]. By analyzing genetic variants—predominantly single nucleotide polymorphisms (SNPs)—GWAS have provided valuable insights into the genetic underpinnings of numerous complex diseases [26], including major depressive disorder [27] and Type 2 diabetes mellitus [28]. GWAS adeptly handle extensive datasets, utilizing SNP arrays or WGS to analyze allele or genotype frequencies across substantial case–control cohorts, enabling the identification of genetic susceptibility loci by correlating SNP frequencies with disease traits in well‐matched populations. This approach considers factors such as ancestry, age, and sex, further enhancing the precision and reliability of GWAS findings.

GWAS have achieved notable success in unveiling new genetic associations, owing to their utilization of substantial sample sizes, often reaching tens of thousands. This extensive scale aids in the discovery of novel genetic associations and the exploration of underlying biological mechanisms. In the context of PN, GWAS have identified genetic variants associated with disease risk and endophenotypes. For instance, a study conducted by Bjornsdottir et al. in 2019 utilized sural nerve conduction data, a widely recognized gold standard for diagnosing PN, to identify a low‐frequency splice–donor variant in *PRPH* associated with reduced sural/sensory nerve action potential [29]. Subsequently, Tao et al. employed a case‐only GWAS design to identify genetic markers associated with specific clinical outcomes in CMT Disease Type 1A (CMT1A) [30]. The study revealed suggestive association signals in four clinical outcomes: difficulty with eating utensils, hearing loss, decreased ability to feel, and CMT neuropathy score. In a more recent study, a Norwegian research group identified two risk loci (rs7294354 in *B4GALNT3* and rs147738081 near *NR5A2*) by conducting the first idiopathic PN focused GWAS using extensive European population‐based health datasets (Trøndelag Health Study and UK Biobank) [31].

The methodological advancements in GWAS have also facilitated the development of polygenic risk scores (PRS), which amalgamate multiple SNP risks into a unified measure of genetic predisposition. While individual SNPs identified via GWAS often have modest effects, their collective impact in PRS can significantly enhance risk stratification, disease prediction, and therapeutic direction, underscoring the clinical relevance of GWAS‐derived data. For further insights into the applications of PRS in clinical and personal contexts, interested readers may refer to comprehensive research and clinical reviews on the subject [32, 33, 34].

Although GWAS have achieved significant success, they are not without limitations. Notable drawbacks include their inability to capture most of the missing heritability and susceptibility to cryptic population stratification [35]. Moreover, significant findings often depend on large cohort sizes, which require additional effort to identify, sequence, and analyze a large population sample. GWAS predominantly identify risk loci rather than causal variants, and many studies depend on genomic imputation based on common SNPs from a reference population [35]. This reliance can obscure the detection of rare or novel variants that are not present in the reference genome.

### 2.2 Targeted Gene Panel Sequencing

As NGS becomes increasingly accessible and affordable in clinical settings, genetic testing has become a routine part of diagnostic evaluation when a genetic variant is suspected to contribute to a patient's condition. Targeted gene panel sequencing is a powerful tool for analyzing specific genes associated with a particular disorder, providing a focused and cost‐effective approach to identifying the genetic causes of CMT disease [36]. In some cases, additional genes are included in these panels to investigate the underlying causes of other clinical features, such as endocrine and metabolic abnormalities, which may co‐occur in patients. This information can confirm a clinical diagnosis and assist in family planning and genetic counseling.

Genetic testing companies and research groups have developed several targeted gene panels to support comprehensive clinical evaluations for CMT. Examples include Invitae's CMT Disease Comprehensive Panel and the Mayo Clinic's Motor and Sensory Neuropathy Panel, which have been widely utilized in clinical practice and research to identify genetic variations linked to CMT [22, 36, 37, 38, 39, 40, 41]. While these panels have proven effective for diagnosing specific subtypes of CMT, their diagnostic yield is often limited. This is largely due to the composition of the studied patient populations and the heterogeneity of the disease.

Despite their utility, targeted gene panels have inherent limitations. They are unable to detect noncoding regions, complex chromosomal rearrangements, or structural variants that might contribute to disease [22, 36]. In some cases, variants within targeted genomic regions may also be missed due to technical limitations. Another significant challenge is that gene panels can quickly become outdated as new disease‐related genes are discovered, limiting their ability to detect variants in emerging candidate genes or genomic regions not included in the original panel design. To address these limitations, gene panels should be periodically updated to reflect the latest advancements in genetic research, ensuring a more comprehensive and up‐to‐date diagnostic tool.

### 2.3 WES

Despite the valuable insights provided by GWAS and targeted gene panel sequencing, their limitations necessitate additional genomic analyses. WES and WGS have become essential for elucidating the genetic architecture of PN. Over the past decade, WES has become a vital tool in research and clinical diagnostics for identifying rare genetic disorders that conventional testing methods often fail to detect.

WES targets exon regions, which make up only 1%–2% of the genome but contain most disease‐causing variants with large effects. This approach achieves high coverage at a lower cost compared to WGS, making it particularly effective for identifying rare coding single nucleotide variations (SNVs) and copy number variations (CNVs) in diverse diseases, both germline [42, 43, 44] and somatic [45, 46]. However, WES is not without its challenges. It does not cover noncoding variants and is prone to mapping issues with short‐read sequencing technologies, which may result in potential false negatives and misdetection of complex structural variations [47].

In the context of hereditary PN, WES has proven invaluable, particularly when previous genetic testing yielded negative results. In 2015, Drew et al. applied WES to a cohort of 110 patients with hereditary PN, including CMT, hereditary sensory neuropathies, and hereditary motor neuropathies, with no genetic diagnosis in the most common PN‐related genes (*PMP22, MPZ*, *GJB1*, *MFN2*, *SPTLC1*, and *SPTLC2*) [48]. They identified nine pathogenic variants in known CMT‐related genes and 12 novel variants of unsure pathogenicity. The following year, Sevilla et al. used WES to uncover a new axonal CMT subtype associated with the *MORC2* p.Arg190Trp variant in both familial and sporadic cases [49]. In 2017, Walsh et al. described a 38% increase in diagnostic yield in a cohort of 55 PN patients using WES [50]. Hartley et al. demonstrated the effectiveness of WES in diagnosing hereditary PN through a molecular basis approach, identifying both reported and novel pathogenic or likely pathogenic genetic variants in a cohort of 50 families with at least one affected individual [51]. Subsequently, in 2020, Senderek et al. utilized WES to investigate a cohort of elderly individuals with unexplained late‐onset axonal neuropathies, revealing disease‐causing variants for CMT and other types of PN [52]. The pathogenic variants detected in this patient cohort significantly advanced the genetic understanding of a fraction of idiopathic axonal neuropathies in the elderly. In another study, Jo et al. explored the detection of CNVs in patients with hereditary PN conditions, such as CMT1A and hereditary neuropathy with pressure palsies [53]. Using three different CNV calling methods, the study accurately pinpointed breakpoints of duplication and deletion events using high‐coverage WES data. Comparing the WES results to the gold standard array comparative genomic hybridization analysis, WES revealed less than 1% difference in breakpoint locations relative to the full length of chromosome 17. This finding demonstrates the feasibility of utilizing WES‐based methods for the rapid, cost‐effective, and accurate identification of large insertions and deletions in patients with hereditary PN.

Clinically, WES continues to facilitate the discovery of novel genetic variants and supports additional diagnostic assessments. For instance, Peddareddygari and Grewal described a patient presenting with numbness and tingling in the feet, with no family history of PN and an unclear etiology for their previously diagnosed condition of chronic inflammatory demyelinating polyneuropathy [54]. Using WES, the clinicians identified a heterozygous variant in the *LITAF* gene, associated with CMT Disease Type 1C (CMT1C). This case illustrates WES's ability to provide a more precise diagnosis for patients with complex clinical manifestations.

### 2.4 WGS

While WES primarily targets the protein‐coding regions, WGS offers comprehensive coverage, including both coding and noncoding regions. This extensive coverage allows WGS to identify genetic variants that may be missed by WES, which is crucial for diagnosing complex genetic diseases like PN.

Short‐read WGS (srWGS) has proven instrumental in diagnosing various forms of PN. As early as 2010, srWGS was applied to a family with a recessive form of CMT with an unknown genetic etiology, leading to the identification of a causative compound heterozygous genotype in the *SH3TC2* gene [24]. This approach not only pinpointed the causative variants but also correlated them with clinical phenotypes, providing actionable diagnostic information. In 2016, Brewer et al. discovered a large 78 kb interchromosomal insertion at chromosome 8q24.3 in the CMTX3 locus that segregated with CMTX3 disease in two families but was absent in neurologically normal chromosomes [55]. In many cases, srWGS has been used to achieve additional diagnostic yield when other genetic testing methods fail to provide definitive results. For instance, the Undiagnosed Disease Network reported that srWGS helped identify a paternally inherited noncoding variant in intron 8 of the *IGHMBP2* gene in a CMT patient [56]. This specific variant, which was not found in any of the existing databases and was missed by WES, was shown to activate a cryptic acceptor site, leading to patient‐specific CMT phenotypes. Such cases highlight the ability of srWGS to detect novel genetic variations contributing to rare and atypical forms of the disease.

Recent advances in srWGS have emphasized its potential in detecting repeat expansions (REs) in PN. Cortese et al. reported that while no recurring nonsynonymous or CNVs were detected in srWGS for patients with cerebellar ataxia, neuropathy, and vestibular areflexia syndrome (CANVAS), a reduced read depth in the *RFC1* repeat region was observed during manual inspection of the sequence reads [57]. This critical observation allowed them to associate *RFC1* pathogenic REs with CANVAS. The initial failure to detect *RFC1* REs directly might be due to sequencing quality limitations and shortcomings in early RE detection tools. However, subsequent studies employing advanced bioinformatic tools analyzed srWGS data from various patient cohorts, not only validating the initial findings but also finding novel pathogenic repeat motifs associated with CANVAS [58, 59]. As sequencing costs continue to decrease, it is anticipated that srWGS will soon become a routine part to aid clinical diagnostics for PN, and to facilitate more precise patient‐specific therapies. Recent studies have demonstrated the efficacy of srWGS in stratifying patients into specific risk groups and guiding treatment decisions, marking a significant advancement in the personalized medicine landscape [60, 61].

However, srWGS only generates reads of 100–300 base pairs, which limits its ability to analyze complex genomic regions, such as those containing large structural variants and highly repetitive or homologous regions [62, 63]. Additionally, srWGS struggles to phase alleles accurately, particularly in compound heterozygous conditions. Whole‐genome long‐read sequencing (lrWGS), offered by platforms like PacBio and Oxford Nanopore Technologies, addresses these limitations by generating reads that span 10–20 kilobases or more [62, 63, 64]. This extended read length enables better characterization of structural variants and improved mapping of complex genomic regions. The Genome in a Bottle Consortium leveraged lrWGS to benchmark structural variants against multiple reference genomes, improving variant calling reliability and providing robust references for genetic haplotypes [65, 66]. lrWGS has also been instrumental in identifying pathogenic structural variants, such as the *DMD* gene that is responsible for Duchenne muscular dystrophy, which traditional srWGS failed to detect [67]. In the PN field, lrWGS has effectively identified noncoding REs in *LRP12*, *GIPC1*, and *RILPL1* genes, with *LRP12* REs being a prevalent cause of CMT [68].

Despite its advantages, lrWGS remains prohibitively expensive, especially in a clinical setting. To enhance the utility of long‐read sequencing (LRS), targeted enrichment at specific genomic regions is employed instead of covering the entire human genome, as in lrWGS. Several subtypes of LRS incorporating this idea include (1) LRS amplicon sequencing (e.g., detecting pathogenic 72.8‐kbp deletion in *BBS9* [69] and tandem repeats in *MUC1* [70]); (2) the Cas9‐assisted targeting of chromosome segments (e.g., characterizing the C9orf72 GGGGCC repeat expansion at the nucleotide level [71]); and (3) targeted LRS on the PacBio or Oxford Nanopore platforms (e.g., identifying several novel pathogenic variants in Werner syndrome [72]).

### 2.5 RNA Sequencing

RNA Sequencing (RNA)‐seq plays a crucial role in understanding PN pathophysiology by examining the transcriptome and offering insights into gene expression patterns and spliced isoforms. Clinical researchers utilize RNA‐seq to analyze blood or tissue samples from patients, extracting valuable transcriptomic data to investigate the underlying mechanisms of their conditions. Bulk RNA‐seq examines the mRNA of the entire tissue samples through steps including RNA extraction, cDNA library preparation, sequencing, and downstream analysis. Techniques such as poly‐A selection and rRNA depletion enhance RNA capture efficiency. The first use of bulk RNA‐seq was in 2006 when Bainbridge utilized massively parallel sequencing by synthesis to profile the mRNA of a human prostate cancer cell‐line LNCaP [73].

In the field of PN, bulk RNA‐seq has been used to study chemotherapy‐induced peripheral neuropathy (CIPN) in patients treated with paclitaxel [74]. Skin punch biopsies from the distal leg of patients with and without CIPN revealed significant gene expression changes in the extracellular matrix, cytoskeleton, and cell‐cycle regulation. Notably, the elevated expression of extracellular matrix‐degrading enzyme MMP‐13 in patients with CIPN was reported, suggesting that extracellular matrix remodeling may play a role in the pathophysiology of the PN in patients with paclitaxel treatment [74]. Another significant application was demonstrated by Ray et al., who used bulk RNA‐seq to characterize human dorsal root ganglia from 50 patients experiencing neuropathic pain [75]. They found enrichment in JUN‐FOS signaling in male patients and increased centromere protein‐coding genes in female patients, highlighting mechanistic sex differences in neuropathic pain [75].

While bulk RNA‐seq can provide researchers with extensive information on gene expression levels that may point toward disease mechanisms and potential therapeutics, there are limitations to its use in a clinical setting. The outcome of bulk RNA‐seq heavily depends on the quality of the patient samples. RNA degrades rapidly due to temperature and RNase enzymes, requiring samples to be harvested and frozen immediately to prevent degradation. Low‐quality RNA could result in 3′‐end bias and misrepresent gene expression levels, affecting the end results. Additionally, disease pathomechanisms are often multifactorial, involving various cell types. Bulk RNA‐seq only provides an overall transcriptome of the entire tissue, which can obscure gene expression changes in rare or less‐dense cell populations, yielding confounding results.

To address these challenges and provide a more granular view of disease pathology, single‐cell and single‐nuclei RNA sequencing (scRNA‐seq/snRNA‐seq) examine transcriptomic information at the individual cell or nuclei levels. In 2009, Tang et al. pioneered NGS‐platform‐based single‐cell transcriptome analysis by characterizing cell‐type heterogeneity from the early developmental stages of a single mouse blastomere [76]. Since then, various algorithms for single‐cell technologies have been developed to reveal complex and rare cell populations, uncover cell‐to‐cell communications, elucidate regulatory relationships between genes, and trace distinct cellular lineages during development [77].

An exemplary application of scRNA‐seq/snRNA‐seq is the injured sciatic nerve atlas (iSNAT) created by Zhao et al., providing important insights into neural tissue degeneration and regeneration [78]. The authors used scRNA‐seq to map out murine naive sciatic nerve, peripheral blood mononuclear cells, and crushed sciatic nerve at various days following injury. Their longitudinal analysis revealed neuroinflammation, immune microenvironments, and intercellular communications through ligand–receptor interactions. Moreover, initiatives like the Human Cell Atlas aim to map every human cell type, yielding transformative insights into the enteric nervous system and offering new markers for neuropathic conditions [79]. The potential of scRNA‐seq extends to clinical applications, as demonstrated by Li et al., who used it to identify potential drug targets for treating diabetic foot ulcers [80].

Despite its transformative potential, scRNA‐seq/snRNA‐seq faces constraints such as high costs, sensitivity to technical variability, sparse datasets, complex data analysis, and a lack of spatial information. Moreover, variations in tissues and cell types during cell/nuclei isolation introduce considerable variability in results. However, emerging technologies like spatial transcriptomics aim to address some of these limitations by adding spatial context to transcriptomic data, allowing researchers to observe gene expression patterns within intact tissues [81, 82, 83]. For instance, Maniatis et al. used spatial transcriptomics to obtain gene expression measurements in mouse spinal cords and postmortem tissue from Amyotrophic Lateral Sclerosis patients, providing insights into the underlying molecular mechanisms [81]. Overall, the expanding toolbox of transcriptomic techniques continues to push the boundaries of our understanding of PN, paving the way for more precise diagnoses and targeted treatments.

### 2.6 Proteomics

While profiling the genome and transcriptome provides key insights into disease causality and susceptibility, previous methods are limited in their ability to characterize post‐translational modifications (PTMs) and the dynamic nature of cellular phenotypes. Proteomic approaches complement genomic studies by establishing a more direct relationship between molecular phenotypes and clinical outcomes. They are particularly valuable in uncovering the phenotypic effects of disease‐associated variants and protein–protein interactions, which are essential for understanding the biological pathways underlying complex disease traits. Furthermore, proteomics can identify protein biomarkers from patient samples, aiding diagnosis, and tracking disease progression or pharmacologic responses to therapeutic interventions.

Proteomic workflows enable direct measurement of protein expression and proteoforms arising from alternative splicing, proteolytic processing, and PTMs. Recent advancements in high‐throughput aptamer and antibody‐based technologies have enhanced the sensitivity and efficiency of protein profiling. For instance, the Proximity Extension Assay by Olink Proteomics allows multiplexing of over 5400 target proteins per sample using NGS in the Olink Explore series or 92 target proteins with high sensitivity using qPCR in the Target series [84, 85, 86]. Similarly, SomaLogic's SomaScan multiplexed proteomic platform uses fluorescently labeled DNA aptamers—short single‐stranded nucleic acids that fold into unique three‐dimensional structures—to selectively bind up to 11,000 molecular targets, facilitating the discovery of novel biomarker candidates in multiple neurodegenerative disorders [87, 88, 89, 90].

A pilot discovery study harnessed unbiased mass spectrometry to identify blood plasma proteins showing differential abundance between 31 IPN patients with severe neuropathic pain and 29 without [91]. Although no significant individual protein candidates were detected after multiple testing correction, Van Doormaal et al. applied multivariate modeling by elastic net analysis to examine the collective associations of multiple proteins [91]. Clustering discovered within the complement and coagulation cascade pathway supports the hypothesized link between immune function and IPN. A systematic review of quantitative proteomics data was used to meta‐analyze blood‐based biomarker concentrations among PN patients across 36 studies, encompassing 4,414 participants and 13 neuropathy diagnostic subtypes, including diabetic PN, CMT, and Guillain–Barre syndrome [92]. The study evaluated the reliability and prevalence of several putative neurodegenerative biomarkers, such as neurofilament light chain (NfL), S100B, brain‐derived neurotrophic factor, and neuron‐specific enolase, highlighting the potential of blood‐based biomarkers in assessing nerve involvement in PN patients. Interestingly, NfL has consistently emerged as a potential biomarker of PN pathogenesis and exhibited utility in assessing disease severity and clinical outcomes [93, 94]. However, the specificity of this broad marker of axon degeneration has been called into question, and further research is required to verify or refute its validity as a clinical tool [95]. Additionally, a recent report used Olink proteomic panels to screen 398 unique proteins in CMT1A patients and found a significant increase in transmembrane serine protease 5 (TMPRSS5) concentration, suggesting its potential as a Schwann cell‐specific biomarker [96]. However, the scarcity of longitudinal studies and significant intrasubject variability of both plasma NfL and TMPRSS5 have prevented their use as outcome measures in clinical trials [94, 97].

The diverse biochemical and structural properties of proteins complicate proteomic profiling, marking a notable disadvantage compared to more standardized DNA‐ or RNA‐based approaches. Challenges in sample preparation, separation, detection, and analysis reduce the sensitivity and reproducibility of proteomic assessment methods. Traditional capture‐based technologies have been widely and successfully employed to mitigate the obstacles imposed by the heterogeneous nature of protein character and abundance [98, 99]. These methods offer highly sensitive and specific protein quantification, but their low throughput and reliance on antibody specificity limit their utility in discovery‐stage studies on protein biomarkers of disease. To address these limitations, novel adaptations such as Luminex and Single Molecule Arrays have been developed, enabling ultrasensitive protein detection in challenging biofluids such as blood plasma and serum [100, 101]. However, despite their high sensitivity and specificity, these methods require antibodies that may introduce batch‐to‐batch variability [102, 103]. Consequently, while proteomic assessments provide invaluable insights into the molecular underpinnings of diseases, their complexity demands meticulous methodological considerations to ensure accuracy and reproducibility in clinical and research settings.

## 3 Discussion

As genomic technologies become more integrated into clinical workflows, their impact on the clinical management of PN is expected to grow. Future research should focus on large‐scale studies to validate the clinical utility of genetic, transcriptomic, and proteomic markers and develop standardized guidelines for their use in clinical practice. Establishing these guidelines is crucial for incorporating these markers into clinical applications. The continued advancement of these technologies promises to deepen our understanding of disease mechanisms, introduce new biomarkers, and unveil novel therapeutic targets, thereby enhancing the precision with which PN is managed.

The rapid evolution of genomics and other complementary technologies heralds a new era in biomedical research and patient care. Below, we explore the potential applications and clinical impacts of these technologies on PN, emphasizing the emerging field of multiomics.

### 3.1 Emerging Multiomic Technologies and Other Techniques

Metabolomics, the comprehensive analysis of metabolites within biological specimens, has emerged as a transformative tool in linking metabolism with neurodegeneration. A pioneering study by Soldevilla et al. in 2017 exemplifies the use of metabolomics to identify biomarkers for subtyping diseases like CMT1A [104]. Furthermore, the works of Auranen et al. and Clark et al. in 2017 and 2021, respectively, have demonstrated how metabolomics can reveal metabolic states, offering essential insights into disease progression and informing therapeutic strategies [105, 106]. These advancements highlight the pivotal role of metabolomics in refining our understanding of disease processes and driving the development of targeted diagnostics and therapies.

Additionally, epigenetics has emerged as a crucial field in understanding the nongenetic factors influencing gene expression. For example, Guo et al. analyzed methylome profiles of sural nerves from Type 2 diabetic patients with diabetic PN and demonstrated DNA methylation as a mechanism for regulating gene expression in diabetic PN [107]. Integrating epigenetic data with genomic, transcriptomic, and proteomic analyses enriches our comprehension of the complex disease pathways at the tissue, cellular, and nuclear levels.

LRS, genome‐wide or targeted, facilitates the identification of various nucleic acid modifications, such as 4‐methylcytosine, 5‐methylcytosine, 5‐hydroxymethylcytosine, *N* ^6^‐methyladenine, and 8‐oxoguanine [108]. This information, coupled with DNA sequence change, could provide invaluable insight into the impact of variants across the genome, facilitating the prioritization of candidate variants. Although its application in PN research is still emerging, LRS holds great promise for increasing diagnostic yields, particularly in unraveling complex structural variants and addressing the elusive heritability that plagues many human diseases [108].

A key strategy in bridging the translational gap in PN research involves the use of patient‐derived fibroblasts to create human induced pluripotent stem cells (iPSCs) or organoid cultures. These models enable detailed studies of disease pathology and the screening of potential therapeutics. For instance, research by Van Lent et al. using iPSC‐derived motor neurons from patients with CMT Type 2 (CMT2) has identified common degenerative hallmarks shared by different CMT2 subtypes, suggesting possible unified treatment approaches [109]. Additionally, the researchers found that mitochondrial dysfunction was a shared phenotype across different CMT2 subtypes, suggesting a potential uniform treatment for CMT2. Using 3D cell culture models of iPSCs and iPSC‐derived human organoid cultures has helped researchers test drugs and treatments for CMT2 subtypes and elucidate the mechanism of PMP22 in CMT1A [106, 110, 111, 112]. Moreover, the use of CRISPR‐based functional genomics in conjunction with iPSC‐derived neurons allows for efficient screening of disease variants and drug testing within robust in vitro models (Table 1).

**TABLE 1:** Pros and cons of multiomics technologies.

Technology	Pros	Cons	References
GWAS	Cheaper than sequencing‐based technologies	Cannot account for all genetic factors contributing to heritability in complex traits Examine only a small subset of the genome, potentially missing important variants	[25, 26, 27, 28, 29, 30, 31, 32, 33, 34, 35]
Targeted gene panel sequencing	Focused and cost‐effective for specific disorders Efficient identification of known pathogenic variants	Limited to known genes, missing novel or rare variants May not detect structural variations outside target regions	[22, 36, 37, 38, 39, 40, 41]
WES	Cost‐effective and covered by insurance High coverage in protein‐coding regions Effective in diagnosing rare genetic disorders	Overlook noncoding genome regions and structural variants outside of exons Fail to capture the full genetic architecture of traits influenced by multiple genes and environmental factor	[42, 43, 44, 45, 46, 47, 48, 49, 50, 51, 52, 53, 54]
srWGS	Comprehensive genome variant identification, including both coding and noncoding, ranging from simple nucleotide variants to complex structural variations	Challenges in the clinical interpretation of noncoding variants Alignment issues associated with short‐read technology	[24, 55, 56, 57, 58, 59, 60, 61]
lrWGS	Detect large structural variants, full‐length isoforms, and repeat expansions Facilitate phased genome assembly and variant calling Recognize various nucleic acid modifications like methylation	Higher per‐base error rates compared to short‐read sequencing Higher cost and more time consuming compared to short‐read sequencing Require substantial computational resources and storage space	[62, 63, 64, 65, 66, 67, 68, 69, 70, 71, 72, 108]
RNA‐seq	Provide information on gene expression, alternative splicing, and isoforms that cannot be captured using WES or WGS Identify differential expressed genes and pathways linked to PN	Sensitive to technical variability and sample quality Sparse datasets	[73, 74, 75, 76, 77, 78, 79, 80, 81, 82, 83]
Proteomics	Detect posttranslational modifications High specificity and sensitivity that enable accurate protein quantification	Some methods restricted by antibody availability and cross‐reactivity Dynamic range limits utility in characterizing complex biofluids	[84, 85, 86, 87, 88, 89, 90, 91, 92, 93, 94, 95, 96, 97, 98, 99, 100, 101, 102, 103]

### 3.2 Clinical Implications of Multiomic Findings in PN

In the United States, genetic testing for CMT disease is typically covered by most insurance plans when recommended by a physician. However, coverage and reimbursement policies can vary significantly across different insurance providers, including Medicare, Medicaid, and private insurers. We recommend a tiered approach for genetic testing that begins with targeted gene panel sequencing as the first‐line method to screen for known genes associated with CMT. This targeted approach has proven to be both cost‐effective and efficient in confirming a clinical diagnosis [113, 114].

For patients receiving a negative result from targeted gene panel sequencing, further testing with WES or WGS should be considered. The choice between WES and WGS may depend on the suspected involvement of complex structural variants or chromosomal abnormalities contributing to disease pathogenesis. In cases where these approaches fail to yield a diagnosis, advanced multiomic technologies, such as transcriptomics or proteomics, can be employed to elucidate molecular mechanisms associated with putative genetic variants.

While these advanced techniques hold great potential, it is crucial to thoroughly evaluate their benefits while taking into account several factors, including cost, payer requirements, the complexity of interpreting variant findings, and the resources needed for effective genetic counseling for patients and families. The implementation of these multiomic approaches must balance their potential benefits with the practical challenges of effectively integrating them into precision medicine.

The integration of genomic data into clinical practice can lead to the adoption of precision medicine approaches in the treatment of PN. Genetic discoveries may help identify patients who would benefit from specific pharmacological agents that target pathways implicated in their disease processes. Understanding the genetic basis of a patient's PN can also help avoid therapies that are less likely to be effective or more likely to cause adverse effects based on their genetic profile.

In cases where PN is part of a broader genetic syndrome, targeted therapies that address the underlying genetic cause can be particularly effective. This approach not only helps in managing the neuropathic symptoms but also addresses other manifestations of the syndrome, offering a holistic approach to patient care.

Integrative analysis of genomic, transcriptomic, and proteomic profiles has the potential to revolutionize diagnosis, prognosis, and treatment of PN. An unclear diagnosis and unknown disease mechanisms pose challenges for clinicians in providing effective treatment, which increases the disease burden on patients. Early and routine sample collection and genomic analysis can identify patient‐specific variants, potentially discovering unknown mechanisms and enabling classification based on the newfound insight. Integrating genomic data with metabolomics profiles, for example, may allow clinicians to identify distinct molecular signatures associated with idiopathic PN, enabling more accurate disease classification and personalized treatment approaches. Establishing efficient methods for quantifying biomarkers will lay the foundation for PN diagnostics using routine equipment at nonspecialized clinical institutions, expediting the evolution of noninvasive modalities for tracking disease trajectory and supporting early intervention.

## 4 Conclusions

The potential for genomic and multiomic studies to reshape the landscape of PN diagnosis and treatment is vast. As the molecular intricacies of PN are decoded and these insights are translated into clinical practice, the prospects for precision medicine—tailoring treatment based on individual genetic and molecular profiles—become increasingly attainable, promising improved outcomes and enhanced quality of life for PN patients.

## Author Contributions

All authors contributed to the literature review, table generation, original draft preparation, and manuscript review and editing. J.C. was responsible for generating the figures. J.C. and Z.T. contributed equally to the writing and execution of the project. S.C.J. conceptualized and supervised the study. S.C.J. and A.H. equally contributed to funding acquisition. All authors have read and approved the final version of the manuscript.

## Conflicts of Interest

The authors report no conflicts of interest.

## Data Availability

Data sharing not applicable to this article as no datasets were generated or analyzed during the current study.
